# Sepsis Patients in Critical Care Units with Obesity: Is Obesity Protective?

**DOI:** 10.7759/cureus.6929

**Published:** 2020-02-10

**Authors:** Charlene Kalani, Tejaswi Venigalla, Janay Bailey, George Udeani, Salim Surani

**Affiliations:** 1 Medicine, Corpus Christi Medical Center, Corpus Christi, USA; 2 Internal Medicine, Corpus Christi Medical Center, Corpus Christi, USA; 3 Internal Medicine, Hunterdon Medical Center, Flemington, USA; 4 Internal Medicine, Texas A&M University, Kingsville, USA; 5 Internal Medicine, Texas A&M Health Science Center, Bryan, USA

**Keywords:** obesity, metabolically-healthy obese, metabolically-unhealthy obese, overweight, morbidly obese, sepsis, morbidity, mortality

## Abstract

Obesity is becoming a global health issue and its prevalence is increasing. It is associated with an increased incidence of illness and sepsis. While obesity is associated with increased morbidity and mortality, obesity has been found to be associated with improvement in mortality outcomes in sepsis when compared to leaner patients, a phenomenon described as an obesity paradox. However, the effect of obesity on mortality in adults requiring treatment for sepsis is unclear. Studies evaluating this effect are inconsistent and there is an increased morbidity still associated with obesity. As well, there are many limitations to these studies confounding interpretation. Future prospective studies minimizing bias and confounding factors are suggested to address this important clinical issue.

## Introduction and background

Obesity is a rapidly growing challenge in the United States and around the globe. Around 70% of adults in the United States are overweight and 35% are obese [1,2]. Due to the high prevalence of obesity, there are an increasing number of obese patients being hospitalized in intensive care units (ICUs). Of the adults admitted to the ICUs, around 25% are overweight, obese, or morbidly obese [3]. Sepsis is the most common cause for admission to the ICU. Sepsis is associated with significant morbidity, mortality, and cost to the healthcare system. In the United States, septicemia was overall the 11th leading cause of death in 2010 according to the Centers for Disease Control and Prevention, and is the most common cause of death in the ICU [4,5]. Globally, there is in-hospital mortality of 15% with sepsis and 25% for severe sepsis [6]. Mortality increases to 40%-50% in patients with complications and in lower-income countries [7,8]. An estimated 50% of patients with sepsis require ICU admission and 17% require intubation [5]. The estimated total cost in the United States for severe sepsis is $24.3 billion dollars per year [9].

Obesity is known to reduce overall life expectancy and is associated with an increased incidence of chronic health conditions such as type two diabetes mellitus, hypertension, heart failure, and coronary artery disease. The World Health Organization defines obesity as a body mass index (BMI) >30 kg/m2 (Table 1) [10].

**Table 1:** The World Health Organization Body Mass Indices Classification

Classification	BMI (kg/m2)
Underweight	<18.5
Normal weight	18.5-24.9
Overweight	25-29.9
Obese class I	30 - 34.9
Obese class II	35 - 39.9
Obese class III/ Morbid obesity	≥40

Several studies have also demonstrated an increased risk of developing infection and sepsis with worse outcomes in infections compared to normal weight patients [11]. Surprisingly, in sepsis, obesity has been revealed in several studies to have better mortality outcomes compared to leaner patients. This unexpected finding has led to the “obesity paradox” where obese patients have lower-than-expected mortality outcomes compared to similar patients who have lower BMIs. This finding can also be found in several different disease states including chronic kidney failure requiring hemodialysis, skin/soft tissue infections, and heart failure [12-15]. The effect of obesity on outcomes with sepsis is still unclear. There have been several studies evaluating the association of obesity and outcomes with sepsis but results are conflicting [3]. We will try to address this issue in this review.

## Review

In a prospective, observational cohort study by Prescott et al., 1,404 severe sepsis hospitalizations were analyzed for in-hospital, 90-day, and one-year mortality in normal weight, overweight, obese, or severely obese patients [16]. The study found that higher BMI was associated with statistically significant improved one-year mortality with obese (odds ratio (OR) 0.59; 95% confidence interval (CI) 0.39-0.88) and severely obese patients (OR 0.46; 95% CI 0.26-0.80) had the lowest mortality rate [16]. While the length of stay in a healthcare facility was greater for obese patients, the study found that average daily utilization was similar with normal and overweight survivors [16]. Obese sepsis survivors utilized more Medicare spending than lower BMIs but the study attributed this to longer survival of the patients [16].

Several retrospective chart review studies evaluated the impact of obesity on survival [17-21]. In a retrospective study by Wochenscher et al., 301 underweight, normal weight, overweight, and obese patients with septic shock were evaluated for outcomes [21]. Patient with BMI >50 kg/m2 were excluded [21]. The study found that overweight and obese patients were independently associated with a decreased risk of ICU death than normal weight patients in a multivariable model but this finding was not statistically significant (OR 0.93; 95% CI 0.86-1.02, p=0.09) [21]. In a second study of 792 septic patients, the study found that survivors had a higher average BMI compared to non-survivors (27.6 vs. 26.3 kg/m2, p=0.03) [19]. However, after adjusting for co-morbidities including age, severity of illness, length of stay, the association was no longer statistically significant (OR 0.52, p=0.03). Similar findings were found in a retrospective, large, multi-center nested cohort study of 8,670 patients with septic shock [17]. The study found obese and very obese patients were associated with lower mortality. However, once again, the results became non-significant once adjusting for co-morbidities and sepsis interventions [17]. A retrospective cohort study of 1,779 patients with sepsis found no significant mortality worsening with obesity (OR 1.11; 95% CI 0.85-1.41, p=0.47) but there was no difference in the hospital length of stay [18]. However, severely obese patients were found to have higher mortality than normal weight patients [18].

In a large meta-analysis including six studies that evaluated the effect of BMI on mortality in sepsis, severe sepsis, or septic shock in patients admitted to ICU, the study found overweight and obese BMIs were associated with significantly decreased mortality after adjusting for baseline variables (OR 0.83; 95% CI 0.75-0.91, p=0.0002 and OR 0.82; 95% CI 0.67-0.99, p=0.04, respectively) [4]. Morbid obese patients were not associated with decreased mortality, but these patients also did not have increased mortality [22]. Table 2 summarizes the details of select studies and their outcome.

**Table 2:** Summary of Select Studies Evaluating Obesity Effect on Mortality in Sepsis BMI = body mass index; CI = confidence interval; LOS = length of stay; N/A = not applicable/not reported; NS = not significant; OR = odds ratio.

Study	No. of patients Study Design	Definitions/ Comparator	Mortality Benefit Yes/No/NS and OR	Mean Length of Stay in Hospital Increased (days)	Length of Intubation	Comments/Notes
Arabi et al^17 ^(2013)	8670 patients with septic shock Retrospective, nested cohort	Comparator: Normal weight Normal weight: 18.5-24.99 kg/m2 Obese: BMI 30-39.99 kg/m2 Very Obese: BMI >40 kg/m2	Crude: YES Obese- OR 0.80, 95% CI 0.66 to 0.97; p=0.02 Very obese- OR 0.61, 95% CI 0.44 to 0.85; p=0.003 Adjusting for baseline characteristics and sepsis interventions: NS Obese- OR 0.80, 95% CI 0.62 to 1.02; p=0.07 Very obese- OR 0.69, 95% CI 0.45 to 1.04; p=0.08	ICU LOS: NS; p=0.30 Obese- 14.7 vs. 12.5 Very obese- 14.3 vs. 12.5 Hospital LOS: YES; p=0.02 Obese- 27.8 vs. 31.8 Very obese- 34.8 vs. 31.8	N/A	There were different approaches to sepsis for obese and normal weight patients
Gaulton et al^18^ (2014)	1,779 patients with presumed sepsis Retrospective cohort	Comparator: Non-obese Non-obese: BMI >18.5 and <30 kg/m2 Obese: BMI >30 kg/m2	NS OR 1.11, 95 % CI 0.85-1.41, p=0.47	Hospital LOS: NS 3.1 vs. 3.83; p=0.45	N/A	ICU location at time of presumed sepsis: Obese 74.2% vs. 69.4% non-obese p=0.04
Kuperman et al^19^ (2013)	792 patients with sepsis Retrospective chart review	Comparator: Normal weight Underweight: BMI <18.5 Normal: BMI 18.5-24.9 kg/m2 Overweight: BMI 25.0-29.9 kg/m2 Obese: BMI 30.0-39.9 kg/m2 Morbidly obese: BMI 40.0-49.9 kg/m2	Underweight: NS OR 1.5, 95% CI 0.67-6.3 Obese: NS OR 0.72 95% CI and p value not reported Morbid obesity: NS OR 0.7, 95% CI 0.12 – 4.2; p = 0.19	NS; p=0.64 Obese: 9.0 ± 8.1 vs. 9.3 ± 8.5 Overweight: 8.8 ± 7.8 vs. 9.3 ± 8.5 Morbidly Obese: 10.2 ± 9.6 vs. 9.3 ± 8.5	N/A	BMI of 27.6 for survivors vs. 26.3 kg/m2 among non-survivors (p=0.03)
Pepper et al^23^ (2019)	55,038 adults with sepsis	Comparator: Normal Weight Normal weight: BMI 18.5-24.9 kg/m2 Overweight: 25.0-29.9 kg/m2 Obese class 1: 30.0-34.9 kg/m2 Obese class II: 35.0-39.9 kg/m2 Obese class III: > 40 kg/m2	YES Adjusted odds ratio (95% CI) Underweight: 1.62 (1.50-1.74) Overweight: 0.73 (0.70-077) Obese class I: 0.61 (0.57-0.66) Obese class II: 0.61 (0.55-0.67) Obese class III: 0.65 (0.59-0.71)	Similar between groups ICU length of stay: 9 days; interquartile range, 6–15 days Hospital length of stay: 8 days; interquartile range, 6–13 days	N/A	Sepsis defined with Sepsis-3 criteria BMI calculated from data on or preceding day of sepsis diagnosis (did not include the weight change after fluid administration) Included death or hospice in mortality outcome Confounders included demographic factors, admission year, hospital-level factors, infection factors, and severity of illness
Prescott et al^17^ (2014)	1404 patients with severe sepsis Prospective, cohort Medicare study Followed patients for 1 year	Comparator: Normal Weight Normal weight: BMI 18.5-24.9 kg/m2 Overweight: BMI 25-29.9 kg/m2 Obese: BMI 30.0-34.9 kg/m2 Severely Obese: BMI >35 kg/m2	YES Obese: OR 0.59; 95% CI, 0.39-0.88 Severely Obese: OR 0.46; 95% CI, 0.26-0.80	Hospital LOS: NS Obese: 11.5 vs. 12.5 Severely Obese: 12.5 vs. 11.5	N/A	Total days in a healthcare facility and Medicare expenditures greater for obese (p< 0.01 for both comparisons) Average daily utilization NS (p=0.44) for overweight and obese survivors Medicare spending NS (p=0.65) for overweight and obese survivors Total function limitations following severe sepsis NS (p=0.64) Increased healthcare use concluded to be due to longer survival
Wacharasint et al 20 (2013)	778 patients with septic shock Retrospective cohort analysis	Comparator: Normal weight Normal weight: BMI <25 kg/m2 Overweight: BMI 25-29.9 kg/m2 Obese: BMI >30.0	28-day mortality: YES Overweight: BMI <25 kg/m2 vs. overweight, p=0.10 Obese: BMI <25 kg/m2 vs. obese, p=0.01 Overweight versus Obese, p=0.2	N/A	No difference in days alive and free of mechanical ventilation (obese 11, overweight 10, BMI <25 kg/m2 6 days; p=0.36	Amount of fluid per body weight significantly lower in overweight and obese patients Day 1 through Day 4; p<0.0001 Days alive and free of renal replacement therapy (obese 25, overweight 24, BMI<25 kg/m2 24 days; p=0.93) Norepinephrine: Obese 0.14, IQR 0.09-0.25 Overweight 0.21, IQR 0.12-0.34 BMI <25 kg/m2 0.26, IQR 0.15-0.44 μg/kg/minute; p< 0.0001 IL-6 significantly lower in obese and overweight compared to <25kg/m2 in early phase of septic shock; p=0.046
Wurzinger et al^21 ^(2010)	301 patients with septic shock Retrospective analysis on prospectively collected database	Comparator: Normal weight Normal weight: BMI 18.5-24.9 kg/m2 Overweight: BMI 25-29.9 kg/m2 Obese: BMI 30.0-39.9 kg/m2 Morbidly Obese: BMI >40 kg/m2	YES Overweight: OR 0.43; 95% CI 0.19-0.98; p=0.04 Obese: OR 0.28; 95% CI 0.08-0.93; p=0.04	ICU LOS: NS; Regression coefficient 0.03, 95% CI -0.15 to 0.21; p=0.74	NS Regression coefficient 0.05 days, 95% CI -0.1 to 0.2; p=0.49	BMI for survivors vs. nonsurvivors: 27 vs. 24; p=0.001 Elevated BMI independently associated with lower frequency of acute delirium (p=0.04) and lower need for ICU re-admission (p=0.001) Elevated BMI associated with higher rate of ICU-acquired urinary tract infections (p=0.02) Need for mechanical ventilation OR 1.02, 95% CI 0.95-1.09; p=0.66

In a recent retrospective analysis by Pepper et al., 55,038 Sepsis-3 criteria septic adult patients were found to have lower short-term mortality in patients with higher body weight, including overweight and obese classes I, II, and III patients, compared to those with normal weight in both unadjusted and adjusted analyses for patient, infection, and hospital-level factors [23]. Hospital and ICU lengths of stays were also similar between the BMI categories. These results further indicate there may be an obesity paradox in sepsis [23]. Despite the possible mortality benefit seen with obesity, there are conflicting results on the potential for increased morbidity associated with obesity, including length of stay in ICU and length of intubation [24,25].

Pathophysiology

The mechanism of the protective function with overweight and obese patients is not understood. One possible reason is that the elevated adipose tissue and associated energy stores may maintain the catabolic state induced by sepsis [26]. However, it is controversial if sepsis truly is a catabolic condition [27].

Another theory is that the systemic inflammatory response with sepsis may be different in obese patients than normal weight patients [28]. Ectopic fat in non-adipose tissue, such as visceral fat, leads to the unhealthy abnormalities that are associated with metabolic syndrome.

Metabolic syndrome is defined when at least three of the following are present [29]:

o Waist circumference >102 cm in males >88 cm in females

o Serum triglycerides >150 or receiving drug treatment for elevated triglycerides

o Serum high-density lipoprotein (HDL) <40 mg/dl in men and <50 mg/dl in women or receiving drug treatment for low HDL levels

o Blood pressure is >130/85 mmHg or receiving drug treatment for hypertension

o Fasting blood glucose is >100 mg/dl or receiving drug treatment for elevated blood glucose

Visceral fat is associated with increased production of cytokines, elevated free fatty acid flux, insulin resistance, and gluconeogenesis [30]. Approximately 50% of overweight adults and 32% of obese individuals may be considered metabolically healthy due to the absence of the metabolic syndrome [31]. As well, about 24% metabolically-obese but normal weight individuals are estimated to be metabolically abnormal where they have a normal BMI but have metabolic syndrome [32]. This syndrome is associated with high risk of developing cardiovascular diseases and increased morbidity and mortality compared to those without the syndrome and may be associated with the chronic disease states of obesity.

A third possible observation for the obesity paradox is that adipocytes could be immunomodulating as obese patients have lower than expected proinflammatory cytokines during severe infection and acute lung injury [20,33]. This is possibly due to chronic inflammation due to obesity. The elevated concentrations of tumor necrosis factor (TNF)-receptors produced by visceral fat, may lead to a decrease in the harmful effects of excessive TNF production during sepsis [34]. Increased lipoprotein levels and adipose tissue may also bind and inactivate harmful bacterial products such as lipopolysaccharide, which augment the septic pathophysiology [3,33]. As well, levels of pro-inflammatory cytokine interleukin-6 is muted in obese patients [20]. Leptin, a cytokine that is produced and stored in adipocytes, is believed to have a protective role in the acute systemic inflammation seen with sepsis by regulating cell-mediated immunity, endothelial activation, and cytokine release. In critically ill patients with sepsis, there is an associated three-fold increased concentration of leptin [22]. Due to the large amount of adipocytes in obese patients, they naturally have increased production and storage of leptin and interleukin-10, another anti-inflammatory cytokine [35]. Elevated levels of leptin have been shown in some studies to have a benefit in mortality but this result is not uniform among all studies [36]. While these responses are thought to be due to the chronic inflammation of obesity, other chronic inflammatory conditions do not have the same mortality benefit seen with obesity in sepsis [37].

Additionally, adipose tissue is associated with increased renin-angiotensin-aldosterone system (RAAS) function. While the excessive RAAS function leads to hypertension associated with obesity, this may be protective during sepsis due to the decreased need for vasopressors, which are associated with many adverse effects [17,20]. 

Limitations

However, there are many limitations identified in the current studies that demonstrate this relationship. There likely were many differences in sepsis interventions in the obese compared to lower BMIs, such as admission status and location and the administration of fluids, vasopressors, and antimicrobial therapy [17,20].

In addition, concerns with obesity hypoventilation syndrome and acute-on-chronic hypercapnic respiratory failure may have led to selection bias of the medical staff caring for overweight or obese patients and more aggressive treatment. Current Surviving Sepsis Campaign (SSC) guidelines recommend noninvasive ventilator strategies first-line in patients with obesity-hypoventilation syndrome, but these methods are often contraindicated due to the underlying acidosis and altered level of consciousness associated with obesity hypoventilation syndrome [38]. The difficulties with intubation and mechanical ventilation due to body habitus of these patients including limited neck mobility, decreased mouth opening, and difficulties with patient positioning may also predispose providers to lower the threshold for ICU admission due to an anticipated difficult airway [3].

There also may be a bias in overestimating the severity of illness in obese patients. Illness severity scores do not usually account for obesity and have not been validated in obese patients [17]. For example, many components of the Acute Physiology and Chronic Health Evaluation (APACHE) II scoring may be affected by obesity and not the acute illness, such as oxygenation. This may have led to an increased level of care for patients in comparison to similar patients with lower BMI. In a study by Gaulton et al., obese patients were more likely to be in the ICU at the time of presumed sepsis in comparison to non-obese patients [18].

Current SSC guidelines recommend fluid resuscitation with an initial fluid bolus of at least 30 ml/kg for patients with severe sepsis and septic shock [38]. However, several studies have shown that over-resuscitation with fluids may be harmful in septic patients and may exacerbate underlying respiratory dysfunction [39-41]. The SSC guidelines do not specify the weight to be used for fluid bolus calculations [38]. However, the studies the SSC guidelines base their recommendations on utilized body weight or a total volume of 2L. Several studies have shown that obese patients receive similar fluid volumes in sepsis to normal BMI with no worsening of outcomes [16,19]. This possibly provides a bias towards survival and better outcomes in obese patients.

In addition, studies have shown a decreased amount of vasopressors used per weight. There is no guidance on whether vasopressors should be dosed based on weight (mcg/kg/min) or not (mcg/min) and both methods are used in practice and clinical trials [16]. In a study by Radosevich and colleagues, obese patients with septic shock required less weight-based doses of norepinephrine and similar total doses compared to non-obese patients with no decrease in mortality [42]. Interestingly, Arabi et al. found that underweight and very obese patients with septic shock had fewer hemodynamic disturbances compared to normal weight patients with similar APACHE II scores and required lower amounts of norepinephrine and epinephrine [17].

SSC guidelines recommend the early administration of antibiotics [38]. However, the volume of distribution for many commonly used antibiotics can be altered in obese patients including vancomycin, macrolides, fluoroquinolones, and most beta-lactams leading to underdosing of these antibiotics in this patient population [43]. Studies have shown obese patients received a lower weight-based dose of antibiotics which may have confounded results [17].

Another limitation is the definition of obesity. The World Health Organization defines BMI using height and weight but this method has many limitations. Namely, BMI does not measure the percentage of body fat to body size, the type of fat, or the location of the body fat and overestimates the amount of fat for those with increased body density (e.g. increased lean muscle weight, high bone density) [44]. Abdominal obesity, one of the markers of metabolic syndrome, has been shown in population-based studies to be an independent and stronger predictor of mortality compared to BMI, especially for non-obese patients [25,45,46]. This finding is consistent even after correcting for BMI [25,45]. As well, inaccuracies with measuring height and weight may have led to erroneous BMI categorization. Aggressive fluid resuscitation in the emergency room can lead to increased BMI measurements. However, despite BMI classifications of body weight having many limitations, other methods of measuring fat such as waist circumference or computed tomography are not routinely performed or often feasible in the Emergency Department or ICU [3].

Additionally, previous studies did not describe the types of nutrition patients received, which may have confounded results. Sepsis may be a catabolic condition and malnutrition upon ICU admission may have attenuated the survival benefit in obese patients [47]. Studies have shown an association with decreased mortality in critically ill patients with enteral tube feedings high in protein [47,48]. As well, enteral tube feeding with excessive amounts of calories was associated with worsened morbidity, especially in obese patients [49]. In a large, retrospective cohort study, Harris and colleagues evaluated critically ill patients on enteral nutrition [28]. The study found that, in the early enteral nutrition subgroup, there were no significant differences in the adjusted hospital mortality between normal weight and overweight or obese patients [38]. As well, underweight patients had decreased to no reduced mortality compared to normal weight patients [28].

Another possible theory is that studies, which adjusted for co-morbid illnesses that are associated with increased BMI, may have decreased or negated the potential detrimental effects of obesity [3].

## Conclusions

Studies have found a paradoxical benefit with obesity in regard to mortality with sepsis. The relationship between BMI and mortality with sepsis may be U-shaped with worsened outcomes for underweight and morbidly obese BMIs. However, results are controversial, data is limited, and results may be due to differences in patient characteristics. BMI is not a good indicator of quantity or quality of fat. Most of those with obese BMIs have increased incidence of metabolic syndrome with is associated with increased overall morbidity and mortality. Despite a possible benefit in mortality, morbidity, including length of stay in the ICU and length of intubation, is increased in obese patients. Large, prospective studies, which use consistent measurements of BMI, adjust for baseline characteristics, and utilize similar sepsis interventions, are required to interpret the possible protective effect of obesity in sepsis. Future prospective studies are suggested to address this important clinical issue.
